# Nutritional and Organoleptic Characterization of Two Quinoa (*Chenopodium quinoa*) Cultivars Grown in Quebec, Canada

**DOI:** 10.3390/foods14132394

**Published:** 2025-07-07

**Authors:** Aria Haiying Huang, Sophie Turcot, Nancy Graveline, Marylène Pelletier, Hugues Plourde, Sébastien Villeneuve, Isabelle Germain

**Affiliations:** 1 Agriculture and Agri-Food Canada, Saint-Hyacinthe Research and Development Centre, Saint-Hyacinthe, QC J2S 8E3, Canada; hai.ying.huang@mail.mcgill.ca (A.H.H.); sophie.turcot@agr.gc.ca (S.T.); nancy.graveline@agr.gc.ca (N.G.); marylene.pelletier2@agr.gc.ca (M.P.); sebastien.villeneuve@agr.gc.ca (S.V.); 2 Faculty of Agricultural and Environmental Sciences, School of Human Nutrition, McGill University Macdonald Campus, Sainte-Anne-de-Bellevue, QC H9X 3V9, Canada; Hugues.plourde@mcgill.ca

**Keywords:** quinoa, nutritional composition, proteins, amino acids, fatty acids, anti-nutrients, saponins, sensory evaluation

## Abstract

Quinoa (*Chenopodium quinoa*) cultivation and consumption have been increasing globally for its nutritional value and agricultural adaptability, with over 120 countries involved in its production. In Canada, quinoa is cultivated as a specialty crop to increase crop diversity and support agroresilience. This study is the first to examine quinoa cultivars grown under northern Quebec conditions and to provide a nutritional and sensory characterization of two Quebec (Canada) varieties (Sweet and Bitter) in comparison to the Bolivian reference cultivar, Royal White. Analyses included proximate composition, amino acids, fatty acids, phenolics, and anti-nutrients. Sensory evaluations involved hedonic and bitterness ranking tests. Bolivian cultivar had higher omega-3 content, while the Quebec cultivars showed favorable protein and lipid profiles, with better lipid health indexes. Protein quality was comparable between the Bolivian and Sweet cultivars. The overall flavor appreciation was similar among twice-brushed Bitter cultivar and Bolivian samples. The Bolivian sample received a better score for texture. Descriptive flavor data support the development of a quinoa flavor lexicon. Notably, total saponins content, commonly used as a bitterness indicator, did not consistently correlate with perceived bitterness, emphasizing the need for a standardized quantification method for cultivar selection and further investigation into other flavor-contributing compounds.

## 1. Introduction

Quinoa is an ancient crop originating from South America, in the Andean region. It has been cultivated for thousands of years. In 2018, a review revealed that 123 countries are now implicated in quinoa production and research [1]. The two biggest producers of quinoa are Peru and Bolivia, with a combined average yield of more than 160,000 tons per year, accounting for over 95% of the total world production in the last decade [2] and covering 74% of global exports [1]. This crop has raised great interest worldwide in the last decade due to its broad genetic variability, adaptability to diverse growing conditions, and tolerance to stress, such as drought, frost, and saline soils [3].

Despite the northern climatic conditions, quinoa production in Canada has surged, reaching 4800 hectares between 2011 and 2016 [4]. Quinoa has emerged as a specialty crop to diversify rotations within cropping systems in Canada. Hence, quinoa can be cultivated sequentially alongside conventional crops on the same plot of land over time. When rotated with crops traditionally grown in continuous approaches, specialty crops, such as quinoa, enhance control of weed, disease, and insect pests while reducing reliance on fertilizer and improving soil physical health and biodiversity [5,6]. Quinoa is recognized for its high water-use efficiency, with a water footprint nearly 60% lower than that of wheat or maize and a water efficiency of protein production (g-proteins per 100 g of grain × 1000/water footprint) of 28.7 [7]. Also, Khakbazan et al. [8] demonstrated that a sequence integrating quinoa into wheat crop systems (wheat–quinoa–field pea–wheat–yellow mustard) in the Canadian prairies yielded one of the highest net returns on investment.

Canadian specialty crop systems require targeted valorization strategies to fully achieve their agronomic, economic, and nutritional potential. Quinoa appears adaptable to northern climates, and various international cultivars have a well-documented nutritional value and culinary versatility. Given protein’s role as a key reference nutrient in plant-based foods, Table 1a reports the protein content of selected Canadian specialty crops, standardized per 100 g of raw product. Mustard seeds have the highest protein content at 26.08 g per 100 g, while raw quinoa contains 14.00 g. For comparison, whole-grain wheat flour and soybeans contain 14.64 g and 10.25 g, respectively. Yet, when adjusted to serving sizes defined by the Canadian Food Guide, 5 mL of mustard seeds provides around 20% of the protein found in a 125 mL serving of cooked quinoa, highlighting the significance of portion size and food form in nutritional evaluations (Table 1b).

Quinoa is generally considered a source of complete protein, with all nine essential amino acids (EAA) and a high content of lysine, which is one of the most limiting amino acids in plant-based diets [9]. Moreover, quinoa is naturally gluten-free and thus beneficial to individuals with gluten intolerance or celiac disease. The potential health-promoting properties of quinoa consumption have been demonstrated by various research across in vitro, animal, and human studies, showing benefits for serum lipid profiles and celiac disease [10,11,12]. A recent review article also discussed quinoa’s potential role as a therapeutic functional food in the prevention and management of metabolic disorders such as obesity, diabetes, cardiovascular diseases, and gut dysbiosis [13].

Regardless of its beneficial nutritional profile, one of the primary challenges with quinoa consumption is its bitter taste. In most quinoa varieties currently cultivated, the bitterness caused by saponins makes them less vulnerable to predator attacks. For humans, the bitterness of quinoa can reduce its sensory quality, making it less appealing in taste. Saponins are a group of anti-nutritional compounds, alongside phytates and tannins. They are mainly found in the seed coat, i.e., the outer layer of the grain, and can be reduced by washing or mechanical (pearling/brushing) removal of seed layers. These processing methods could alter the nutritional content and quality of the grains depending on the intensity and duration [14,15].

According to Codex Alimentarius published by FAO and WHO [16], the maximum limit of saponins for quinoa must be below 0.12%. This percentage is derived from Bolivian standards, which were established using the afrosymetric (foam) method. Varieties are classified as sweet or bitter based on whether their saponin content exceeds the determined threshold before processing. Thus, to respect the current standards and increase consumers’ acceptability, Quebec producers (Canada) are breeding and processing quinoa to decrease saponin content to reach a more palatable product.

To date, no research has been published on quinoa crops grown in the northern conditions of Quebec, Canada. Globally, data on the flavor attributes of quinoa is also limited. Therefore, this study aimed to offer a comprehensive characterization of two cultivars of Quebec-grown white quinoa by (1) measuring and comparing the nutritional profiles and anti-nutrient contents with a Bolivian white quinoa; (2) investigating the organoleptic profile of the cultivars, including the relationship between saponins content, mechanical brushing levels, and bitterness taste perception; and (3) performing hedonic sensory evaluation as well as establishing a descriptive lexicon.

## 2. Materials and Methods

### 2.1. Origin of Quinoa Samples

Six white quinoa samples were studied: (1) a Quebec Bitter cultivar in three different conditions (non-brushed (BQNB), mechanically brushed once (BQB1), and mechanically brushed twice (BQB2)) and (2) a Quebec Sweet cultivar in two different conditions (non-brushed (SQNB) and mechanically brushed once (SQB)). Quebec-grown cultivars were provided by NovaQuinoa (L’Assomption, Quebec, Canada). Finally, as control, a commercially available quinoa from Bolivia, i.e., Royal White (BRW), obtained from a local retailer (L’Assomption, Quebec, Canada; Lot: 19059), was used. Both Bitter and Sweet cultivars were harvested in summer 2022, stored in industrial grain silos, and processed through an identical grain cleaning procedure before brushing. Quinoa samples with different degrees of brushing were treated as distinct samples, as brushing can affect the nutritional profile.

Following agricultural processing standards, a three-step sequential separation and cleaning protocol was used: (1) a gravity separator differentiated particles based on weight and density, leveraging slope, vibration, and controlled air flow; (2) mechanical screening followed, wherein the grain passed through four gyratory screens and two sieve plates of varying mesh sizes to eliminate residual foreign matter; and (3) an optical sorting system removed contaminants of similar size and weight but differing in color—primarily radish and mustard seeds. This multi-stage cleaning cycle was typically repeated twice to ensure thorough purification before the final brushing stage.

Batches of 20 kg of each sample were acquired in November 2023 and stored in airtight polyethylene containers in walk-in refrigerated room at 4 °C until further analyses.

### 2.2. Determination of Proximate Composition

Raw quinoa grains were finely milled into flour using IKA A11 Basic Analytical Mill and stored at 4 °C until analysis. The proximate analyses for moisture, ash, total lipid, and protein were performed using the methods described by AOAC [17]. Nitrogen conversion was caried out using a 6.25 factor for protein determination. A Megazyme assay kit (Bray, Ireland) was used for total dietary fiber (TDF) determination in duplicate. Total proteins and lipids content were measured in six replicates. All other analyses were performed in triplicate. Available carbohydrate was estimated by subtracting the mean value of measured parameters using Equation (1).

Carbohydrate (g) = 100 − [moisture (g) + ash (g) + lipid (g) + protein (g) + TDF (g)](1)

### 2.3. Amino Acid Content by HPLC

All amino acids were determined by an HPLC system with a fluorescence detector (FID). First, samples were hydrolyzed as described in the AACC International Methods for amino acids (method 07-01.01) with slight modifications. Briefly, 20 mg of ground sample was weighed in 50 mL Pyrex test tube, and then, 4 mL 6N HCl containing 0.1% phenol was added. Hydrolysis was conducted under nitrogen for 24 h at 110 ± 1 °C. After hydrolysis, HCl was removed under a stream of N2 at room temperature. Given that tryptophane was destroyed by acid, an alkaline hydrolysis was performed as described by Yust et al. [18].

Hydrolysates were dissolved in a volume of buffer appropriate for amino acid analyzer and filtrated on 0.45 µm PVDF filters before injection onto the HPLC-FID system (1260, Infinity, Agilent Technologies Inc. Santa Clara, CA, USA). Amino acids were separated using the Agilent AdvanceBio AAA LC column (4.6 × 100 mm, 2.7 µm) with a precolumn derivatization and quantified with the fluorescence detector. Norvaline was used as an internal standard. The chromatographic conditions used for the amino acids analysis with HPLC-FID were described in the “How-To” Guide from Agilent Technologies Inc. The amino acids content is expressed in mg/g protein. Determinations were performed in duplicate. The chemical method allowed for the determination of 17 amino acids: alanine, arginine, aspartic acid, glutamic acid, glycine, histidine, isoleucine, leucine, lysine, methionine, phenylalanine, proline, serine, threonine, tryptophane, tyrosine, and valine.

### 2.4. Fatty Acids Content by GC-MS

First, lipids were extracted in VELP cups, dissolved in 5 mL hexane, and transferred to a 10 mL amber vial using a glass pipette. The hexane extract was stored at −20 °C until methylation. To prepare the methyl esters, 500 µL of the lipid extract was transferred into a screw-cap glass tube. Next, 2 mL of 0.01 M NaOH in methanol was added, and the mixture was heated at 65 °C for 10 min before cooling. Subsequently, 3 mL of BF3-methanol was added, and the mixture was heated again at 65 °C for 10 min before cooling. To facilitate phase separation, 2 mL of 20% (*w/w*) NaCl solution and 1 mL of hexane were added, and the mixture was inverted to mix, then centrifuged at 1000× *g* for 2 min at 25 °C. The upper phase was recovered and transferred to a 1.8 mL amber vial, sealed, and placed in the autosampler tray. Gas chromatography analysis was performed using an Agilent 6890 J&W DB-Fast FAME Capillary GC Column (30 m × 0.25 mm i.d., df = 0.25 µm) (Agilent Technologies Canada Inc., Mississauga, ON, Canada). The instrument was equipped with a split/splitless injector, maintained at 250 °C with a split ratio of 80:1 and an injection volume of 1 µL. The initial oven temperature was 50 °C, followed by a ramp of 30 °C/min to 194 °C, then further increased at 5 °C/min to 240 °C. Hydrogen was used as the carrier gas at a flow of 1.2 mL/min in a constant pressure mode. The flame ionization detector (FID) was operated at 280 °C. Peaks were identified by comparison of their retention times with those of standards (37-component FAME mix; Supelco (Millipore Sigma Canada LTD, Oakville, ON, Canada)) [19]. Using software HP Chemstation (Version B.01.01., Agilent Technologies Inc., Santa Clara, CA, USA), peak areas were computed, and percentages of the fatty acids were obtained as %weight, with coefficient of variation < 5%.

Based on the percentages of FA obtained, the atherogenicity index (AI), the thrombogenicity index (TI), and the hypocholesterolemic/hypercholesterolemic ratio (H/H) were calculated using the following Equations (2)–(4) [20].

AI = [C12:0 + (4 × C14:0) + C16:0]/(MUFA + n3 PUFA + n6 PUFA)(2)

TI = (C14:0 + C16:0 + C18:0)/[(0.5 × MUFA) + (0.5 × n6 PUFA) + (3 × n3 PUFA) + (n3 PUFA)/(n6 PUFA)](3)

H/H = (C18:1 + C18:2 LA + C18:3 ALA)/(C12:0 + C14:0 + C16:0)(4)

### 2.5. Total Phenolic Content and Antioxidant Activity

Total phenolic content was measured using the Folin–Ciocalteu method described by Nickel et al. [15], with modifications. Briefly, 2.5 g of ground quinoa was added to 25 mL of acidified methanol (0.1% HCL *v/v*) overnight at −20 °C overnight for extraction. Then, 1 mL of centrifuged extract was reacted with Folin–Ciocalteu reagent and sodium carbonate solution (7% p/p). Gallic acid was used as the standard calibration curve. Absorbance was measured using UV–vis spectrophotometer (Thermo Scientific GENESYS 10, Madison, WI, USA) at 760 nm. Results are expressed in mg gallic acid equivalent per 100 g of dry sample (mg GAE/g dw).

Antioxidant activity was measured using the qualitative screening method 2,2-diphenyl-1-picrylhydrazyl (DPPH) free radical scavenging technique [21] for relative comparison between samples. The assay described by Nickel et al. [15], with modifications, was used. Briefly, 2.5 g of quinoa flour was mixed with 25 mL of methanol by vertical agitation Rotator Genie (Scientific Industries) for 2 h, then stored in the dark for 24 h. The extract was centrifuged at 3000× *g* for 10 min. A solution of DPPH at 6.45 × 10^−5^ M was prepared using methanol (control absorbance reading at 0.600 ± 0.01 at 517 nm against methanol). Directly into a polystyrene cuvette, 1 mL of DPPH solution was added to 100 µL of extract supernatant or methanol as control. Cuvettes were immediately covered with a lid and left to stand 20 min in the dark. The sample and control solution absorbance were measured using the Genesys UV–vis Spectrophotometer (Thermo Scientific GENESYS 10, Madison, WI, USA) at 517 nm against methanol as the blank. The antioxidant activity, expressed as % inhibition, was then calculated using the following Equation (5) for each sample:

% inhibition = (Control absorbance − Sample absorbance) × 100/Control absorbance(5)

### 2.6. Anti-Nutritional Compounds Content

Phytic acid was determined by enzymatic procedure using the Megazyme assay kit (K-PHYT) (Ireland).

The tannins content was measured by spectrophotometry [22,23]. Briefly, 0.5 g of quinoa flour was added to 40 mL of 10% ethanol in a covered glass beaker. The mixture was then heated in a boiling water bath for 2 h under constantly stirring with a magnetic stir bar for tannins extraction. The extract was cooled and centrifuged at 3000× *g* for 30 min. The supernatant was collected and filtered using 25 mm GD/X 0.45 µm Whatman Syringe Filter. Next, 2.5 mL of 10% Folin–Ciocalteu reagent was added to 2.5 mL of extract. After 5 min, 5 mL of sodium carbonate solution (7% *w/w*) was added to the solution and left to stand for color development at room temperature for 30 min. The solution was centrifuged at 3000× *g* for 10 min before absorbance reading using Genesys UV–vis Spectrophotometer (Thermo Fisher) at 760 nm. The test was performed in duplicate, with a coefficient of variation < 0.2%. The tannin content was estimated using tannic acid standard curve, expressed in g/100 g flour dw.

Total saponins content (TSC) was determined using spectrophotometry, following the method by Nickel et al. [15] with slight modifications. In brief, 1.0 g of whole quinoa seeds was added to 10 mL of 50% ethanol in centrifuge tube and left to macerate for 72 h at room temperature for saponins extraction. After maceration, the mixture was vortexed for 30 s and centrifuged at 3000× *g* for 5 min before collecting the supernatant fraction. The extract was diluted in a ratio of 1:5 with 50% ethanol. Next, 3.5 mL of Liebermann–Burchard reagent (16.7% acetic anhydride in H_2_S0_4_) was added to 1.0 mL of diluted extract, vortexed immediately, and left for 30 min at room temperature for color development. The reaction was performed in triplicate. The absorbance was measured at 528 nm using the Genesys UV–vis Spectrophotometer (Thermo Fisher). Saponins contents were estimated using a Saponin Quillaja extract (S4521; CAS No. 8047-15-2) standard curve (duplicate), expressed as g/100 g quinoa sample dw. The standard afrosymetric estimation of saponins content in quinoa was performed based on published protocols [24,25].

### 2.7. Sensory Evaluation

Four of the six quinoa samples were selected for sensory evaluation: BRW, BQB1, BQB2, and SQB. The two non-brushed samples were excluded from sensory evaluation because non-brushed samples would not have met the current commercial standard of whole-grain quinoa for human consumption. Moreover, a significant bitter taste in BQNB was detected by preliminary testing.

Unrinsed samples were cooked in ultrafiltered water at a 1:4 grain-to-water ratio, determined through preliminary tests to ensure adequate water for evaporation without over-diluting the samples. Cooking time (min) was determined as BRW = 10, BQB1 = 17, BQB2 = 13, and SQB = 14. Samples were deemed cooked when the germ had separated from the grain. A small amount of excess water was drained after cooking and the quinoa left to cool for 15 min before portioning in reusable amber glass containers, covered using plastic screw lid, and kept in the refrigerator at 4 °C. Samples were prepared one day before each session. Samples were left at room temperature for one hour before being served to the panel. Ultrafiltered water and unsalted crackers were provided to the panelists before the session and between samples to remove the potential lingering taste of the previous sample.

First, a sensory evaluation and descriptive characterization of the selected samples was conducted by 69 untrained assessors recruited during a public event at the research center. The sample size was determined in accordance with ISO 11136:2014, which recommends a minimum of 60 participants for consumer testing [26]. Voluntary participants were asked to evaluate each coded sample in three parts. Samples were presented in random order. First, they were asked to determine the overall appreciation of the flavors found in the sample using a 9-point hedonic scale (1—extremely unpleasant; 2—very unpleasant; 3—unpleasant; 4—fairly unpleasant; 5—neither pleasant nor unpleasant; 6—fairly pleasant; 7—pleasant; 8—very pleasant; 9—extremely pleasant). Then, they were asked to identify aroma and taste attributes from the sample using the following provided list of descriptive terms adapted from Wu et al. (2017) [27]: starchy, butter, woody (e.g., toothpick; wooden stick), caramel, roasted (e.g., bread toast), herbaceous (e.g., freshly cut grass), nutty, bean-like (e.g., cooked kidney beans), earthy (e.g., fresh mushroom), foreign/non-food, and other. Participants were asked to specify if they selected foreign/non-food or other. There was no limit on the number of terms that could be selected. Lastly, participants determined texture appreciation using a 9-point hedonic scale (same as for flavors appreciation). All three parts of the questionnaire were answered for each sample before moving on to the next one. Participants were allowed to retaste samples.

Secondly, bitterness ranking tests were conducted by panels of semi-trained volunteer assessors recruited among staff members from the Saint-Hyacinthe Research and Development Center of Agriculture and Agri-Food Canada or students working at the facility. The minimum number of assessors was set to be 15 [28]. The first panel consisted of 16 judges, while the second included 21 judges. All judges passed a preliminary bitterness sensitivity screening test using N-Propylthiouracil (PROP) test paper during the recruitment stage. Training included one session of 45 min, during which they were presented with two bitter solutions of 0.05% and 0.08% caffeine (30 mL), one astringent solution of 1 g/L tannic acid (30 mL), one acid solution of 0.05% citric acid (30 mL), and two food products with well-known bitter taste (i.e., arugula and 90% dark chocolate). During the ranking test session, the panelists were asked to rank the level of bitterness from the lowest (1) to highest (4) level among coded samples presented in random order. No equality was permitted.

The protocol was approved by the Agriculture and Agri-Food Canada’s Human Research Ethics Committee, and all participants signed an informed consent form before the start of the experiment.

### 2.8. Statistical Analysis

For nutritional analysis, results are expressed as mean value ± standard deviation. One-way analysis of variance (ANOVA) was performed as well as Tukey’s test for post hoc pairwise comparison. Differences were considered significant at *p* < 0.05. Data analysis and processing were performed using SAS Studio (Version 3.81, SAS Institute Inc., Cary, NC, USA).

Results from the 9-point hedonic scales (flavors and texture appreciation) were analyzed using ANOVA and Tukey’s test for pairwise comparison (*p* < 0.05). Ranking tests were analyzed using the Friedman’s test for differences at 5% significance level. All sensory analysis data were collected and processed using Compusense (V24.0.30233, Compusense Inc., Guelph, ON, Canada).

## 3. Results and Discussion

### 3.1. Proximate Composition

Proximate composition results, expressed as % ± standard deviation when applicable, can be found in Table 2. Compared to Quebec quinoa cultivars, BRW quinoa presented significantly lower moisture (7.77 ± 0.02%), ash (2.05 ± 0.01 g/100 g dw), protein (13.53 ± 0.31 g/100 g dw), and lipid (6.60 ± 0.2 g/100 g dw) contents. Significantly higher moisture content was measured in SQB (12.32 ± 0.10%). It is worth noting that the retail packaging of the BRW used in the present study indicated that the quinoa was “pre-washed”, suggesting that the grains underwent a drying process, which may explain its lower moisture content compared to raw Quebec-grown quinoa samples, which underwent a different cleaning process. SQNB presented with significantly higher contents of ash (2.8 ± 0.04 g/100 g dw) and protein (15.99 ± 0.34 g/100 g dw). The lipid content among the Québec cultivars ranged from 7.08 ± 0.23 to 7.42 ± 0.08 g/100 g dw, all exceeding the BRW value.

Overall, Quebec-grown quinoa had a higher nutritional content than the Bolivian variety, mostly in protein, lipid, and ash contents. Based on previously reported ranges for quinoa, protein and fiber content varied greatly, with reported values ranging from 9.10 to 18.8 g protein/100 g dw [29,30] and from 1.00 to 14.1 g fiber/100 g dw [29,31]. These values align with this study’s findings, with Quebec-grown quinoa’s protein content slightly over the average value of 14%, ranging from 14.33 to 15.99 g/100 g dw, and a TDF content that ranged between 11.58 and 14.18 g/100 g dw, which was close to the upper limit of the reported range. A recent article published in 2024 on Canadian-grown quinoa cultivars in two of the Prairies Provinces (Manitoba and Saskatchewan) showed similar values for proximate composition except for fiber content, which was much higher [32]. Since fiber content was estimated by difference in their study rather than measured directly, the resulting values may not be directly comparable. For quinoa cultivated in Ontario, the crude fat content ranged from 6.03% to 6.74% [33], which is slightly lower than the values reported here, where levels ranged between 7.08 and 7.42%. These variations reinforce the impact of environmental factors such as climate, soil, and post-harvest handling on quinoa’s nutritional composition [34,35,36].

While the primary aim of this study was not to investigate the effects of mechanical brushing on grain samples, the procedure was found to exert a notable influence on the proximate composition. These observations align with and reinforce findings reported in prior studies [14,37]. Briefly, in both Sweet and Bitter cultivars, brushing led to a significant reduction in ash, while lipid content slightly increased. Protein levels showed a significant decline in the Sweet cultivar (SQNB vs. SQB), decreasing from 15.99 ± 0.34 to 14.33 ± 0.20 g/100 g dw, respectively, but remained stable in the Bitter cultivar, with no significant differences between BQNB, BQB1, and BQB2.

### 3.2. Total Phenolic Content (TPC) and Total Antioxidant Capacity (TAC)

TPC and TAC results are shown in Table 2. Among the samples analyzed, BQB1 and BQB2 displayed the highest total phenolic content (TPC), with values of 88.60 and 93.43 mg GAE/100 g dw, respectively. BQNB had the lowest TPC (73.45 mg GAE/100 g dw). There was no significant difference in TPC between BRW, SQNB, and SQB, with values ranging from 73.45 to 75.92 mg GAE/100 g. Previous studies reported similar values in a range from 71.7 mg GAE/100 g in Bolivian quinoa [38] to 97.69 mg GAE/100 g in quinoa grown in Brazil [15]. For total antioxidant capacity (TAC), the Bitter and Sweet non-brushed samples grown in Quebec, namely SQNB and BQNB, demonstrated the higher relative % inhibition at 64% and 53%, respectively, compared to BRW and other brushed samples. While direct comparison with previous findings is restricted by methodological differences, these findings provide valuable insights into antioxidant activity using the DPPH free radical scavenging as a relative qualitative comparison between samples. For a more comprehensive quantification of TAC, oxygen radical absorbance capacity (ORAC), 2,2′-azino-bis(3-ethylbenzothiazoline-6-sulfonic acid (ABTS) [39], and ferric ion reducing antioxidant parameter (FRAP) [40] could be measured. Future studies could consider integrating a multi-assay approach, particularly in research primarily focused on the assessment of antioxidant activity.

It is interesting to note that the brushing of the Bitter cultivar increased TPC significantly, but there was no difference between the first and second brushing levels. In contrast, a study that investigated the effect of processing found that there was a reduction in TPC after polishing from 31.6–105.85 mg GAE/100 g in raw grains to 26.31–67.86 mg GAE/100 g in polished grains, namely in the Puno and Titicaca varieties, respectively [37]. This study also used the Folin–Ciocalteu method. In another study [41], which measured both the free and bound phenolic compounds using HPLC, their content was also found to be decreased after pearling by 21.5% and 35.2%, respectively. Therefore, the increase observed in the Bitter cultivar in this study after the brushing process does not align with previous findings. This discrepancy may be attributed to the limitations of the Folin–Ciocalteu method, which lacks specificity and can be influenced by the overall food assessed. The presence of other reducing agents—such as certain amino acids and their derivatives (e.g., cysteine, glutathione, etc.), metal ions, and reducing sugars—can interfere with the measurement [21]. The brushing process might have made these compounds more available and thus increased the reaction with the Folin reagent. Future research could use more specific and selective methods such as HPLC/UPLC or GC approaches to study separate phenolic compounds.

### 3.3. Anti-Nutrients (Phytates and Tannins)

Table 2 shows the content in anti-nutrients in raw quinoa grain samples. Saponins content will be discussed in Section 3.6. Phytate and tannin contents were detected at low concentrations (<0.5%) across all samples, ranging from 0.17 to 0.31 g/100 g dw and 0.20 to 0.29 g/100 g dw, respectively. No statistically significant differences were observed among samples for phytate levels, and no clear trend could be identified in tannin content distribution. Previous studies reported similar content in phytic acid, ranging from 0.2 to 0.8 g/100 g, while tannins ranged from 0.02 to 0.03 g/100 g [31], which is 10 times lower compared to samples in this study. Tannins in quinoa are rarely investigated in the current literature; further studies could consider its contribution to astringency in the flavor profile.

### 3.4. Amino Acids Composition

Samples were analyzed for amino acids in duplicates (Table 3). Leucine, lysine, phenylalanine, and tyrosine were the most abundant essential amino acids across all quinoa samples analyzed. Lysine is reported to be the limiting AA in grains, and people following a vegan diet could be more likely to develop lysine inadequacy [9]. Quinoa, therefore, stands out from other cereal grains and could be a good complementary plant-based protein source due to its higher lysine content. Tryptophan had the lowest content at around 8 mg/g of protein. With the EAA content, amino acid scores (AAS) were calculated based on the reference patterns from FAO’s 2013 report on protein quality evaluation, and the limiting AA for each sample was determined based on the lowest score. Valine was found to be the limiting amino acid with the lowest AAS for BRW and SQNB, whereas methionine had the lowest AAS in BQNB, making it the limiting AA.

While both methionine and valine are essential, methionine is often more limiting in plant-based proteins, especially legumes and cereals, while valine is less commonly limiting in plant-based proteins. This is therefore not in accordance with previous studies on EAA profile in quinoa [30,44]. It was reported that AA content can vary by genotype, growing location [45], and seasons [30]. Amino acids were not analyzed in the article published on Canadian quinoa cultivars [32,33], so a comparison could not be made with quinoa cultivated in closer geographic locations. Leucine was identified as the limiting EEA among the 100 samples of quinoa grown in Washington in the United States [45].

In order to better evaluate the samples’ protein quality, the protein digestibility-corrected amino acid scores (PDCAAS) were also calculated for each sample based on the in-vitro protein digestibility percentage previously reported in quinoa [43]. Despite the fact that digestible indispensable amino acid score (DIAAS) was recommended as a more accurately measurement of protein digestibility compared to PDCAAS [46], there is not yet any reported DIAAS value for quinoa protein, with insufficient data on individual amino acids’ digestibility in quinoa to calculate this score [47]. Results showed that BRW and SQNB had very similar PDCAAS values at 0.784 and 0.786 respectively, while BQNB had a lower PDCAAS at 0.627, suggesting a lower protein quality compared to BRW and SQNB. Overall, quinoa has a lower PDCAAS score than soy (0.91) but still better compared to other cereal grains, such as wheat (0.45) and oat (0.57) [48]. An in vitro study using amino acid assay and animal feeding experiments demonstrated that the digestibility of the protein in quinoa is comparable to that of other high-quality food proteins [44].

### 3.5. Fatty Acid

The fatty acid (FA) composition results (expressed as % of total fatty acids ± SD) are shown in Table 4. For fatty acids not detected in a sample group, a value of 0 was assigned for statistical analysis based on consistent absence across all replicates, except for C:20 and C:22, which were not analyzed due to content non-detected in most samples.

Linoleic, oleic, palmitic, and α-linolenic acids were the predominant fatty acids, accounting for approximately 95% of the total FA content across all cultivars. Among the samples, BRW had the lowest content of linoleic acid (48.47%) and the highest content of oleic (27.88%) and α-linolenic acid (9.93%). In contrast, SQNB had the lowest content of α-linolenic acid (5.92%) and the highest content in palmitic acid (13.61%). The effect of brushing on the FA composition seemed inconsistent for both quinoa cultivars analyzed. For the sweet cultivar, the brushed sample (SQB) compared to the non-brushed (SQNB) had higher contents of oleic acid (21.44 vs. 20.86%), linoleic acid (54.80 vs. 53.94%), and α-linolenic acid (7.24 vs. 5.92%) but a lower content of palmitic acid (11.00 vs. 13.61%). For the Bitter cultivar, the FA content variations were also inconsistent amongst the three samples with different levels of brushing (BQNB, BQB1, and BQB2). Despite statistically significant differences, variations between samples remained relatively small, generally within ~2.5% across brushing levels. Overall, the results of the main FA measured in this study align with the previously reported range of values as well [33,49].

Eicosenoic and erucic acids were present in all samples (1.34–1.49% and 1.12–1.74%, respectively). Stearic and trans-linoleic acids were low (0.66–1.18% and 1.1–1.28%) in Quebec samples but absent in BRW. Trace amounts (<1%) of behenic acid were present in SQB and BQB2 as well as arachidic acid in SQB.

Based on the FA composition, the sums of FAs and the ratios of PUFA/SFA and n-6/n-3 were calculated. More than half of the FAs were part of the PUFA group since linoleic acid had the highest content amongst all FAs. MUFA’s proportion ranged from 22.1% to 30.5%, and SFA contributed <15% to the total FA composition. SFA content was highest in SQNB (14.79 ± 0.01%) and lowest in BRW (11.52 ± 0.09%), while MUFA was most abundant in BRW (30.09 ± 0.46%). PUFA was highest in BQB1 (65.59 ± 0.03%), contributing to its most favorable PUFA/SFA ratio (5.33 ± 0.01%). The n-6/n-3 ratio was most balanced in BRW (4.88 ± 0.01%), whereas SQNB exhibited the highest ratio (9.33 ± 0.01%), indicating a less favorable omega–FA balance. It has been suggested that the n-6/n-3 ratio’s ideal range should be between 1:1 to 5:1, while Westernized diets are characterized by high consumption of n-6, which raises the n-6/n-3 ratio to a range of 10:1 to 20:1, which increases the risk of developing inflammatory diseases as obesity [50].

Given certain limitations associated with FA group ratios as the sole indicators of lipid quality, the atherogenicity (AI) and thrombogenicity (TI) indices developed by Ulbritcht and Southgate [51] were also calculated, as they offer a more accurate assessment of fatty acid composition concerning health outcomes [52]. SQB had the lowest AI (0.126 ± 0.000) and highest H/H ratio (7.592 ± 0.007), suggesting superior cardiovascular benefits. In contrast, SQNB had the highest AI (0.160 ± 0.000) and TI (0.257 ± 0.000) and the lowest H/H ratio (5.931 ± 0.004), indicating a less desirable lipid profile. While BRW stands out for its low SFA and most balanced n6/n3 ratio, SQB had best overall lipid profile based on health indexes. Finally, the brushed quinoa samples (SQB, BQB1, and BQB2) showed better lipid profiles than the non-brushed ones (SQNB and BQNB).

### 3.6. Sensory Evaluation and Saponins Content

#### 3.6.1. Hedonic Assessment and Descriptive Vocabulary

The hedonic sensory evaluation was conducted with a panel of untrained assessors (n = 69), comprising 51% females and 49% males, aged between 19 and 76 years.

- Overall Flavor Appreciation: Mean hedonic scores (on a 9-point scale) for overall flavor were as follows: BRW = 6.23, SQB = 5.78, BQB1 = 5.61, and BQB2 = 5.81. These values fall between the hedonic descriptors “neither pleasant nor unpleasant” (score of 5) and “pleasant” (score of 7). Pairwise comparisons revealed that BRW was rated significantly higher than SQB and BQB1 (*p* < 0.05) but was not significantly different from BQB2. No significant differences were observed between SQB and BQB1;
- Texture Appreciation: Mean texture scores were BRW = 6.77, SQB = 6.29, BQB1 = 6.24, and BQB2 = 6.01, indicating ratings between “fairly pleasant” (score of 6) and “pleasant” (score of 7). BRW received the highest texture rating and was significantly different from all other samples (*p* < 0.05). No significant differences were found among SQB, BQB1, and BQB2.

In the hedonic assessment of cooked quinoa samples, BRW and BQB2 were equally appreciated for their overall flavor by the judges, but BRW still had better texture appreciation compared to the other three samples. In fact, a very recent correlation study [53] on the influence of the nutritional composition on the organoleptic quality of cooked quinoa showed that quinoa varieties with high content in starch, moderate content in fat and moisture, and low content in protein, fiber, and ash were better appreciated and had better cooking quality. This is in agreement with our findings, where BRW had lower protein, ash, and fiber and higher carbohydrates content compared to other samples. It was also reported that higher protein in quinoa contributes to higher firmness and adhesiveness and a gummier, chewier texture in cooked grains [54].

In addition to proximate composition, the seed coat layer was reported to be positively related to hardness, cohesiveness, gumminess, and chewiness [54]. However, in the samples used in this study, BQB1 and BQB2—where BQB2 had reduced coat layer with an additional brushing cycle compared to BQB1—did not show differences in subjective texture appreciation. Another possible factor influencing texture is starch composition, particularly total amylose content, which was reported to be positively correlated with texture parameters [55]. Findings from these previous studies provide indirect but valuable insights into the texture parameters worth investigating in the pursuit of a desirable quinoa texture profile.

**Figure 1:**
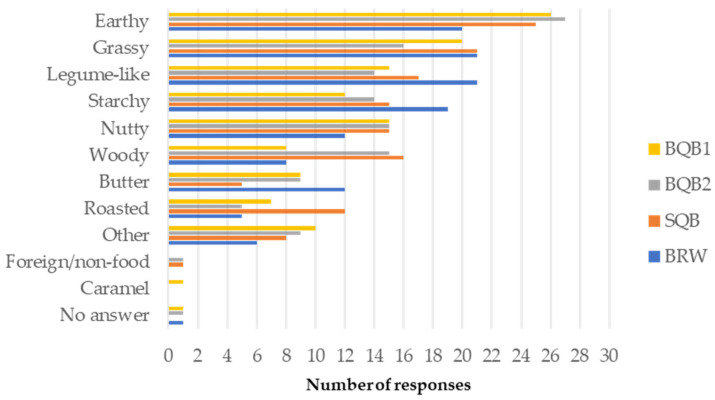
Flavor descriptive attributes associated with cooked quinoa samples (BQB1, Bitter quinoa brushed once; BQB2, Bitter quinoa brushed twice; SQB, Sweet quinoa brushed; BRW, Bolivian Royal White); responses given by untrained judges (n = 69).

- Descriptive Sensory Attributes: Aromas and flavor profiles of products such as beer [56], Canadian maple products [57], and coffee [58] have been systematically characterized and represented through flavor wheels to support sensory quality assessment across diverse commercial products. Nevertheless, no standardized descriptive lexicon currently exists for the sensory evaluation of pseudocereals, including quinoa. Figure 1 presents the frequency of flavor descriptors associated with each sample. The most frequently selected terms for cooked quinoa were earthy, grassy, legume-like, and starchy. Compared to the Québec samples, BRW was more commonly associated with starchy and legume-like notes and less frequently with nutty notes. In contrast, SQB, BQB1, and BQB2 were more frequently described as earthy. BQB2 was perceived as the least grassy, while both BQB2 and SQB were more often associated with woody notes. Finally, SQB was uniquely characterized by a roasted flavor. These results support previous findings reporting a significant impact of quinoa variety on aroma attributes such as caramel, nutty, buttery, grassy, earthy, and woody [27]. It was also noted in that study that consumers preferred a grassier aroma and a less woody aroma. However, this does not align with the results of the present study since BRW and BQB2 received similar overall appreciation despite BQB2 having less frequent mention of grassy and more of woody aroma. Other flavors more frequently identified in BQB2 compared to BRW, such as earthy and nutty, may have contributed to its favorable rating. Repeated sensory evaluations and expert judges could help in providing descriptors for these new Canadian crops.

#### 3.6.2. Saponins Content and Bitterness Perception

The afrosymetric estimation method of threshold saponin concentration for bitterness in quinoa, first developed in 1990, has been a screening test commonly used by agronomists in the field [25]. The limit of 0.12% saponins content suggested with the afrosymetric method is also indicated in the Codex Alimentarius Standard for quinoa (CXS 333-2019, amended in 2020) [16]. However, the official analysis method of saponins content has not been published in the Recommended Methods of Analysis and Sampling (CXS 234-1999, last amendment in 2023). Various analytical methods had been used in previous studies on quinoa saponins content, including major differences in extraction conditions, separation, and quantification techniques, making cross-study comparison difficult.

In this study, TSC was measured using both UV–vis spectrophotometry and the afrosymetric method. In previous studies that used spectrophotometry as method of quantification, values ranged from 0.40 to 4.93% [59], 0.66 to 3.09% [60], and 2.48 to 3.63% [15] as compared to our findings, which ranged from 1.95 to 6.59% (See Table 2 for individual values), with BQNB having the highest value (6.59 ± 0.69%). TSC was overall significantly lower for BRW, SQNB, and SQB, and there were no significant differences among them. Brushing significantly reduced saponin levels for the Bitter cultivar, with BQB1 and BQB2 showing lower TSC compared to BQNB. However, there were no statistically significant differences between BQB1 and BQB2 using the spectrophotometry method. Nevertheless, all samples exceeded the 0.12% limit by far, even for the BRW and Sweet cultivar samples, which should theoretically respect the 0.12% limit. In addition to fact that this limit was determined based on the foam method, this discrepancy could also be explained by the fact that the standard of saponins extract available and used (CAS No. 8047-15-2) was derived from another plant species, Saponin Quillaja.

**Figure 2:**
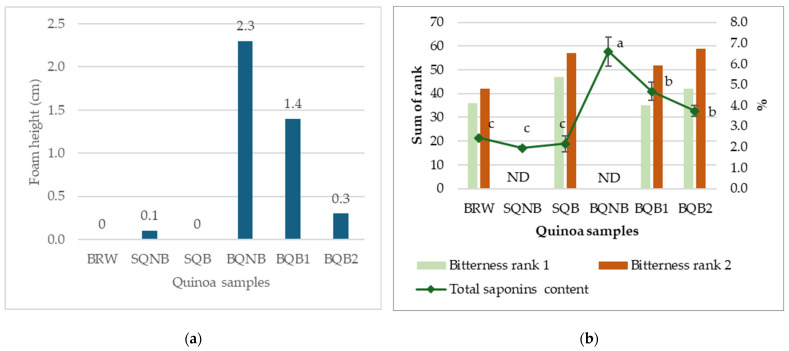
(**a**) Saponins content estimated in quinoa samples using afrosymetric method, expressed in foam height (cm), where samples with a foam height of 1.0 cm or less can be considered as “sweet” [25]. (**b**) Comparison between bitterness rankings sensory tests and total saponins content (measured using UV–vis spectrophotometry method). ND, not determined for bitterness ranking test. (Samples: BRW, Bolivian Royal White; SQNB, Sweet quinoa non-brushed; SQB, Sweet quinoa brushed; BQNB, Bitter quinoa non-brushed; BQB1, Bitter quinoa brushed once; BQB2, Bitter quinoa brushed twice). Different letters represent significant differences (*p* < 0.05).

In contrast, results from the afrosymetric method (shown in Figure 2a) corresponded better to the 0.12% saponins limit. They also demonstrated that the second brushing of the bitter cultivar further decreased TSC to an acceptable level (<1.0 cm foam height) for the quinoa to be considered as “sweet”. Subsequently, sensory evaluation was performed to assess and validate the relationship between saponins content and bitterness perception, especially given the inconsistent TSC results.

Two independent bitterness ranking tests were conducted in sensory evaluation. In test 1, 21 judges were recruited, where 5 of them did not pass the PROP screening test for bitterness perception ability. Thus, there were 16 participants in the panel (11 females and 5 males) who were aged between 22 and 59 years. In test 2, 25 judges were recruited, and 4 of them did not pass the PROP test, forming a panel of 21 judges (17 females and 4 males) aged between 21 and 59 years. The sum of ranks for BRW, SQB, BQB1, and BQB2 were 36, 47, 35, and 42 in test 1 (χ^2^ = 3.53, *p* = 0.32) and 42, 57, 52, and 59 in test 2 (χ^2^ = 4.94, *p* = 0.18), indicating no significant differences in perceived bitterness in both tests. Figure 2b represents a visual comparison between the results from both bitterness ranking tests (bars) and the total saponins content measured by UV–vis spectrophotometry method (diamond markers). While there are significant differences (*p* < 0.05) in TSC, represented with different letters, there is no significant difference in the sum of rank between the four tested samples with the Friedman test (*p* < 0.05). SQNB and BQNB were not assessed by the judges for their bitterness.

Remarkably, based on the above findings, saponin content did not correlate consistently with the human sensory perception of bitterness. BQB1 exceeded the limit in the foam method, while its bitterness remained undistinguishable from other quinoa samples that were considered as “sweet”. Saponins are a large group of compounds with two main groups, namely triterpenoid and steroidal, and many sub-groups with different chemical properties [61]. Although mainly responsible for bitter taste, some saponins have sweetening effects [62,63]. *Chenopodium quinoa* is known to be one of the most saponins-rich species in the Amaranthaceae family, with over 80 saponins identified [64], but there are still very scarce data on their specific role in bitter taste. One recent study investigated five specific phytochemicals contributing to bitterness in quinoa, with very different dose-over-threshold factors, including four saponin compounds and one kaempferol derivative [65]. It is also worth noting that another report investigating bitterness in quinoa, based on electronic tongue response, suggested that saponins may contribute to the umami taste through observed binding with umami receptors, while flavonoids like rutin and kaempferol could contribute more to the bitterness [66]. Alkaloids are also generally known to cause bitterness in plant foods; a study found that alkaloids in faba bean can activate the bitter taste receptors [67], but they have not yet been identified in quinoa to be related to bitterness. In that sense, a “sweet” variety with low TSC could also have other unpleasant off-flavors caused by the presence of other phytochemicals making them equally “bitter” even when the saponins levels are lower. In fact, this suggests that TSC might not be an accurate reflection of the bitterness perception if saponins indeed have different bitterness thresholds.

Moreover, flavor perception is complex and can be influenced by multiple factors, including the presence of proteins and fat/water interface [68]. In addition to saponins and other phytochemicals, it is known that FA could also contribute to the bitter taste in foods, especially PUFAs, which are more prone to oxidation [69]. For instance, linoleic and alpha-linolenic acids were found to have the lowest bitter taste thresholds in emulsion and contributed to the off-taste problems in soybean lecithins [70]. However, a study showed that the addition of linoleic acid lowered the bitterness sensitivity in adults [71]. In our quinoa samples, there were significant differences in the FA composition, but due to small sample size, correlation analysis was not possible.

Overall, these findings highlight the need for a standardized method for saponins quantification corresponding with the established limit as well as a broader investigation into the interactions between food matrix components and flavor-active compounds that influence quinoa’s organoleptic profile.

## 4. Conclusions

This study provided a comprehensive characterization of two Canadian (Quebec) quinoa cultivars (Sweet and Bitter) under different brushing conditions compared to a Bolivian cultivar. Quebec cultivars were found to have an excellent nutritional profile, exhibiting advantageous protein and lipid compositions and offering more favorable lipid health indices than the Bolivian cultivar despite the latter’s higher omega-3 fatty acid content. Protein quality in terms of AA composition was comparable between the Bolivian and Sweet cultivars.

Despite superior texture ratings for the Bolivian sample, overall liking was comparable to the twice-brushed Bitter cultivar, highlighting the suspected sensory benefits of the brushing treatment. Descriptive flavor insights contribute to the development of a quinoa flavor lexicon for industry use. Although saponin content, as assessed by the foam test, is commonly used as a surrogate measure of bitterness, this study found no consistent correlation between taste and total saponin content, highlighting the need for a standardized saponin quantification method and further exploration of other flavor-contributing compounds.

As taste is a major factor driving consumer acceptance and purchase intent, future research should explore broader nutrient–flavor interactions. Breeding programs should integrate both nutritional and sensory traits to support quinoa’s development as a high-value crop in Canada, with key benefits for agroecosystem resilience and food security.

## Figures and Tables

**Table 1:** (**a**) Nutritional values of selected crops (per 100 g), ranked by protein content. (**b**) Nutritional values of ready-to-eat (per serving size) foods, ranked by protein content.

(**a**)								
**#**	**Crops**	**Food Code ^1^**	**Quantity**	**Energy (kcal)**	**Proteins (g)**	**Carbs (g)**	**TDF (g)**	**Fat (g)**
1	Mustard seeds	192	Per 100 g	508	26.08	28.09	12.2	36.24
2	Lentils, raw (pink)	4869		358	23.91	63.10	10.8	2.17
3	Canary seeds	N/A		383	21	60	6.5	6.5
4	Sunflower seeds	2526		585	20.78	20.00	8.6	51.46
5	Rapeseeds ^2^	N/A		642	18.6	5.4	8.5	44.1
6	Flaxseeds	4528		534	18.29	28.88	27.3	42.16
7	Whole-wheat flour	4500		369	14.64	68.51	8.9	3.24
**8**	**Quinoa, seeds**	**4495**		**368**	**14.12**	**64.16**	**6.9**	**6.07**
9	Soybean, edamame	6217		110	10.25	8.58	4.8	4.73
(**b**)								
**#**	**Crops**	**Food Code ^1^**	**Portion Size ^4^**	**Energy (kcal)**	**Proteins (g)**	**Carbs (g)**	**TDF (g)**	**Fat (g)**
1	Lentils, cooked (pink)	5918	175 mL/179 g	190	13.67	32.41	5.9	1.19
2	Soybean, Edamame	6218	125 mL	100	8.91	8.14	4.3	4.26
3	Canary seeds ^3^	N/A	45 mL/30 g	115	6.3	18	1.95	1.95
4	Sunflower seeds	2527	60 mL/32 g	189	6.27	7.81	3.6	16.17
5	**Quinoa, cooked**	**5917**	**125 mL**	**117**	**4.30**	**20.82**	**2.7**	**1.88**
6	Whole-wheat bread	4500	1 slice	90	3.74	15.97	2.2	1.06
7	Flaxseeds (linseed)	4528	15 mL/7 g	38	1.30	2.05	1.9	2.99
**8**	Mustard seeds	192	5 mL/3 g	17	0.88	0.95	0.4	1.22
9	Rapeseed (canola oil)	421	15 mL/14 g	126	0.00	0.00	0.0	14.19

Note: ^1^ Data from Canadian Nutrient File, except for rapeseeds and canary seeds; ^2^ INRA CIRAD AFZ, Feed materials; ^3^ Canary Seed Development Commission of Saskatchewan; ^4^ as suggested by Canadian Food Guide when available.

**Table 2:** Proximate composition, total phenolic content (TPC), total antioxidant capacity (TAC), total saponins content (TSC), phytates, and tannins content in raw quinoa grains.

Parameters (Mean ± SD)	Quinoa Grains					
	BRW	SQNB	SQB	BQNB	BQB1	BQB2
Moisture (n = 3), %	7.77 ± 0.02 e	11.48 ± 0.09 b	12.32 ± 0.10 a	10.80 ± 0.08 c	10.61 ± 0.08 c	9.69 ± 0.16 d
Ash (n = 3), g/100 g dw	2.05 ± 0.01 e	2.80 ± 0.04 a	2.70 ± 0.02 b	2.65 ± 0.02 b	2.47 ± 0.04 c	2.29 ± 0.04 d
Protein (n = 6), g/100 g dw	13.53 ± 0.31 c	15.99 ± 0.34 a	14.33 ± 0.20 b	14.74 ± 0.57 b	14.96 ± 0.34 b	14.40 ± 0.55 b
Lipid (n = 6), g/100 g dw	6.60 ± 0.20 d	7.08 ± 0.23 b	7.42 ± 0.08 a	7.15 ± 0.12 abc	7.16 ± 0.19 abc	7.08 ± 0.11 bc
Total dietary fiber (n = 2), g/100 g dw	10.82 ± 0.81	14.18 ± 0.72	13.89 ± 1.09	11.92 ± 0.30	12.74 ± 0.78	11.58 ± 0.60
Carbohydrate, g/100 g dw	59.23	48.47	49.34	52.74	52.06	54.96
TPC (n = 3), mg GAE/100 g dw	75.92 ± 2.55 b	73.45 ± 1.73 b	74.48 ± 2.92 bc	69.95 ± 2.69 c	88.60 ± 0.82 a	93.43 ± 1.52 a
TAC (n = 3), % inhibition	48.87 ± 2.00 c	64.39 ± 0.73 a	45.42 ± 1.25 cd	53.27 ± 0.21 b	45.28 ± 1.56 cd	44.29 ± 1.63 d
TSC (n = 3), g/100 g dw	2.44 ± 0.04 c	2.15 ± 0.38 c	1.95 ± 0.09 c	6.59 ± 0.69 a	4.67 ± 0.43 b	3.73 ± 0.25 b
Phytates (n = 3), g/100 g dw	0.26 ± 0.03	0.31 ± 0.10	0.30 ± 0.07	0.28 ± 0.08	* 0.17 ± 0.02	0.20 ± 0.02
Tannins (n = 2), g/100 g dw	0.22	0.29	0.24	0.23	0.22	0.20

Note: Samples included the following: BRW, Bolivian Royal White; SQNB, Sweet quinoa non-brushed; SQB, Sweet quinoa brushed; BQNB, Bitter quinoa non-brushed; BQB1, Bitter quinoa brushed once; BQB2, Bitter quinoa brushed twice. Carbohydrate content was calculated by difference of mean values. Tannins content had negligible SD values (<0.001) with a coefficient of variation < 0.5%. Different letters in the same row represent significant differences (*p* < 0.05). * n = 2.

**Table 3:** Essential amino acids (EAA) content, amino acids scores (AAS), and protein digestibility-corrected amino acid score (PDCAAS) of raw quinoa grains.

EAA mg/g Protein	Quinoa Grains			FAO Reference Pattern *	AAS		
	BRW	SQNB	BQNB		BRW	SQNB	BQNB
Histidine	27.98 ± 0.46	29.94 ± 0.19	25.24 ± 0.15	16	1.750	1.869	1.575
Isoleucine	37.58 ± 1.17	37.25 ± 1.74	33.96 ± 1.12	30	1.253	1.240	1.133
Leucine	63.18 ± 2.01	63.65 ± 2.16	57.56 ± 1.47	61	1.036	1.044	0.944
Lysine	61.04 ± 2.96	59.20 ± 3.00	45.44 ± 1.04	48	1.271	1.233	0.946
Methionine	24.31 ± 1.04	25.86 ± 0.63	18.52 ± 1.04	23	1.057	1.126	**0.804**
Phe + Tyr	70.67 ± 2.15	73.83 ± 2.56	64.82 ± 1.47	41	1.724	1.800	1.580
Threonine	35.28 ± 0.60	36.69 ± 0.94	32.83 ± 0.85	25	1.412	1.468	1.312
Tryptophan	8.10 ± 2.52	8.76 ± 0.54	7.93 ± 0.53	6.6	1.227	1.136	1.197
Valine	40.19 ± 1.00	40.26 ± 1.45	36.57 ± 0.92	40	**1.005**	**1.008**	0.915
				PDCAAS	0.784	0.786	0.627

Note: * 2013 report; values for children (>3 y), adolescents, and adults [42]. Methionine is presented on its own since cysteine was not determined. Phe + Tyr = Phenylalanine + Tyrosine. Limiting amino acid score is indicated in bold. PDCAAS calculated based on quinoa protein digestibility of 78% [43]. Samples included the following: BRW, Bolivian Royal White; SQNB, Sweet quinoa non-brushed; BQNB, Bitter quinoa non-brushed.

**Table 4:** Fatty acids (FA) composition expressed in % ± SD, main groups of FA, and health indexes (AI, atherogenicity index; TI, thrombogenicity index; H/H, hypocholesterolemic/hypercholesterolemic ratio) of raw quinoa grains.

Parameters	Quinoa Grains					
	BRW	SQNB	SQB	BQNB	BQB1	BQB2
Palmitic acid (C16:0)	11.52 ± 0.09 d	13.61 ± 0.01 a	11.00 ± 0.04 e	11.91 ± 0.02 b	11.65 ± 0.03 c	11.46 ± 0.03 d
Stearic acid (C18:0)	nd	1.18 ± 0.01 a	0.70 ± 0.01 c	0.74 ± 0.01 b	0.66 ± 0.01 d	0.688 ± 0.005 c
Oleic acid (C18:1n9c)	27.88 ± 0.18 a	20.86 ± 0.04 c	21.44 ± 0.08 b	19.52 ± 0.06 d	19.16 ± 0.01 e	19.52 ± 0.08 d
Linoleic acid (C18:2n6c)	48.47 ± 0.31 e	53.94 ± 0.05 d	54.80 ± 0.14 c	56.82 ± 0.09 b	57.36 ± 0.03 a	56.93 ± 0.09 b
Trans-linoleic acid (C18:2n6t)	nd	1.284 ± 0.002 a	1.10 ± 0.01 c	1.13 ± 0.01 b	1.14 ± 0.01 b	1.101 ± 0.005 c
α-linolenic acid (C18:3n3)	9.93 ± 0.07 a	5.92 ± 0.01 e	7.24 ± 0.01 b	7.08 ± 0.02 c	7.09 ± 0.01 c	6.69 ± 0.01 d
Arachidic acid (C20:0)	nd	nd	0.39 ± 0.01	nd	nd	nd
Eicosenoic acid (C20:1n9)	1.46 ± 0.01 b	1.464 ± 0.004 b	1.49 ± 0.01 a	1.34 ± 0.01 d	1.405 ± 0.003 c	1.39 ± 0.01 c
Behenic acid (C22:0)	nd	nd	0.51 ± 0.01	nd	nd	0.45 ± 0.01
Erucic acid (C22:1n9)	1.122 ± 0.004 c	1.74 ± 0.03 a	1.47 ± 0.03 b	1.47 ± 0.03 b	1.53 ± 0.01 b	1.50 ± 0.03 b
SFA	11.52 ± 0.09 d	14.79 ± 0.01 a	12.46 ± 0.26 bc	12.65 ± 0.02 b	12.31 ± 0.03 c	12.60 ± 0.05 bc
MUFA	30.09 ± 0.46 a	24.06 ± 0.06 b	24.40 ± 0.04 b	22.33 ± 0.09 c	22.10 ± 0.01 c	22.41 ± 0.11 c
PUFA	58.40 ± 0.37 e	61.14 ± 0.05 d	63.14 ± 0.15 c	65.02 ± 0.11 b	65.59 ± 0.03 a	64.71 ± 0.09 b
PUFA/SFA ratio	5.07 ± 0.01 b	4.13 ± 0.00 c	5.07 ± 0.10 b	5.14 ± 0.02 b	5.33 ± 0.01 a	5.14 ± 0.01 b
n-6	48.47 ± 0.31 e	55.23 ± 0.05 d	55.89 ± 0.15 c	57.94 ± 0.09 b	58.50 ± 0.02 a	58.03 ± 0.09 b
n-3	9.93 ± 0.07 a	5.92 ± 0.01 e	7.24 ± 0.01 b	7.08 ± 0.02 c	7.09 ± 0.01 c	6.69 ± 0.01 d
n-6/n-3 ratio	4.88 ± 0.01 f	9.33 ± 0.01 a	7.72 ± 0.02 e	8.19 ± 0.02 d	8.25 ± 0.01 c	8.68 ± 0.02 b
AI	0.130 ± 0.001 d	0.160 ± 0.000 a	0.126 ± 0.000 e	0.136 ± 0.000 b	0.133 ± 0.000 c	0.132 ± 0.000 cd
TI	0.166 ± 0.001 e	0.257 ± 0.000 a	0.189 ± 0.001 d	0.206 ± 0.001 b	0.200 ± 0.001 c	0.201 ± 0.000 c
H/H	7.492 ± 0.014 b	5.931 ± 0.004 f	7.592 ± 0.007 a	7.006 ± 0.016 e	7.178 ± 0.024 d	7.255 ± 0.008 c

Note: nd, not detected. Different letters on the same row represent significant differences (*p* < 0.05). C20:0 and C22:0 were not analyzed for statistical differences because they were not detected in most samples. Abbreviations: BRW, Bolivian Royal White; SQNB, Sweet quinoa non-brushed; SQB, Sweet quinoa brushed; BQNB, Bitter quinoa non-brushed; BQB1, Bitter quinoa brushed once; BQB2, Bitter quinoa brushed twice; SFA, saturated FA; MUFA, mono-unsaturated FA; PUFA, poly-unsaturated FA.

## Data Availability

The original contributions presented in this study are included in the article. Further inquiries can be directed to the corresponding author(s). The raw data supporting the conclusions of this article will be made available by the authors upon reasonable request.
