# Correction: Huang et al. Nutritional and Organoleptic Characterization of Two Quinoa (*Chenopodium quinoa*) Cultivars Grown in Quebec, Canada. *Foods* 2025, *14*, 2394

**DOI:** 10.3390/foods15020184

**Published:** 2026-01-06

**Authors:** Aria Haiying Huang, Sophie Turcot, Nancy Graveline, Marylène Pelletier, Hugues Plourde, Sébastien Villeneuve, Isabelle Germain

**Affiliations:** 1 Agriculture and Agri-Food Canada, Saint-Hyacinthe Research and Development Centre, Saint-Hyacinthe, QC J2S 8E3, Canada; hai.ying.huang@mail.mcgill.ca (A.H.H.); sophie.turcot@agr.gc.ca (S.T.); nancy.graveline@agr.gc.ca (N.G.); marylene.pelletier2@agr.gc.ca (M.P.); sebastien.villeneuve@agr.gc.ca (S.V.); 2 Faculty of Agricultural and Environmental Sciences, School of Human Nutrition, McGill University Macdonald Campus, Sainte-Anne-de-Bellevue, QC H9X 3V9, Canada; Hugues.plourde@mcgill.ca

**Additional Copyright**

In the published publication [1], there was an error regarding the copyright. The copyright should be added: “© His Majesty the King in Right of Canada, as represented by the Minister of Agriculture and Agri-Food Canada 2025. Licensee MDPI, Basel, Switzerland. This article is an open access article distributed under the terms and conditions of the Creative Commons Attribution (CC BY) license.” The authors state that the scientific conclusions are unaffected. This correction was approved by the Academic Editor. The original publication has also been updated.
